# The efficacy of Protected Mealtimes in hospitalised patients: a stepped wedge cluster randomised controlled trial

**DOI:** 10.1186/s12916-017-0780-1

**Published:** 2017-02-07

**Authors:** Judi Porter, Terry P. Haines, Helen Truby

**Affiliations:** 1 Department of Nutrition, Dietetics and Food, Monash University, 264 Ferntree Gully Road, Notting Hill, Victoria 3168 Australia; 2 Allied Health Research Office, Eastern Health, Box Hill, Victoria 3128 Australia; 3 Allied Health Research Unit, Monash Health, Clayton, Victoria 3168 Australia

## Abstract

**Background:**

Protected Mealtimes is an intervention developed to address the problem of malnutrition in hospitalised patients through increasing positive interruptions (such as feeding assistance) whilst minimising unnecessary interruptions (including ward rounds and diagnostic procedures) during mealtimes. This clinical trial aimed to measure the effect of implementing Protected Mealtimes on the energy and protein intake of patients admitted to the subacute setting.

**Methods:**

A prospective, stepped wedge cluster randomised controlled trial was undertaken across three hospital sites at one health network in Melbourne, Australia. All patients, except those receiving end-of-life care or not receiving oral nutrition, admitted to these wards during the study period participated. The intervention was guided by the British Hospital Caterers Association reference policy on Protected Mealtimes and by principles of implementation science. Primary outcome measures were daily energy and protein intake. The study was powered to determine whether the intervention closed the daily energy deficit between estimated intake and energy requirements measured as 1900 kJ/day in the pilot study for this trial.

**Results:**

There were 149 unique participants, including 38 who crossed over from the control to intervention period as the Protected Mealtimes intervention was implemented. In total, 416 observations of 24-hour food intake were obtained. Energy intake was not significantly different between the intervention ([mean ± SD] 6479 ± 2486 kJ/day) and control (6532 ± 2328 kJ/day) conditions (*p* = 0.88). Daily protein intake was also not significantly different between the intervention (68.6 ± 26.0 g/day) and control (67.0 ± 25.2 g/day) conditions (*p* = 0.86). The differences between estimated energy/protein requirements and estimated energy/protein intakes were also limited between groups. The adjusted analysis yielded significant findings for energy deficit: (coefficient [robust 95% CI], *p* value) of –1405 (–2354 to –457), *p* = 0.004. Variability in implementation across aspects of Protected Mealtimes policy components was noted.

**Conclusions:**

The findings of this trial mirror the findings of other observational studies of Protected Mealtimes implementation where nutritional intakes were observed. Very few positive improvements to nutritional intake have been identified as a result of Protected Mealtimes implementation. Instead of this intervention, approaches with a greater level of evidence for improving nutritional outcomes, such as mealtime assistance, other food-based approaches and the use of oral nutrition support products to supplement oral diet, should be considered in the quest to reduce hospital malnutrition.

**Trial registration:**

Australian New Zealand Clinical Trials Registry: ACTRN12614001316695; registered 16th December 2014.

## Background

Malnutrition is highly prevalent in patients in westernised countries [1, 2], with associated adverse clinical outcomes including increased length of stay, morbidity and mortality and decreased quality of life. This nutritional decline has been attributed to “a continuum of inadequate intake and/or increased requirements, impaired absorption, altered transport, and altered nutrient utilization” ([3], p. 730). Malnutrition contributes to spiralling healthcare costs. In the UK in 2015, Elia et al. [4] estimated that the expenditure related to malnutrition was £19.6 billion, or about 15% of the total health and social care expenditure. Critically this report noted that small fractional cost savings would translate to large absolute savings, and this supports the rationale to further the effort to identify measures to prevent and treat malnutrition in hospitals.

One key contributing factor to malnutrition is the low level of patient food and drink intake relative to their nutritional needs [5, 6]. This is not a new issue, as the philosophy integral to reserving mealtimes to provide the uninterrupted opportunity to eat was first described by Florence Nightingale in 1860: “To leave the patient’s untasted food by his side, from meal to meal, in hopes that he will eat it in the interval is simply to prevent him from taking any food at all” and “…it ought to be a rule WITHOUT ANY EXCEPTION WHATEVER, that no one shall bring business to him or talk to him while he is taking food, nor go on talking to him on interesting subjects up to the last moment before his meals, nor make an engagement with him immediately after, so that there be any hurry of mind while taking them” [7].

Malnutrition has a multi-factorial aetiology [8], and as such, systems thinking has been applied in the modern practice setting. One such patient-centred systems change has been the adoption of Protected Mealtimes [9]. This initiative aims to “protect mealtimes from unnecessary and avoidable interruptions, providing an environment conducive to eating, assisting staff to provide patients/clients with support and assistance with meals, placing food first at mealtimes” [9]. In practice, Protected Mealtimes implementation has a substantial impact on staff routines, including those of medical staff, as implementation includes ceasing ward rounds, drug rounds and general practitioner visits during eating occasions. The National Health Service (NHS) has supported Protected Mealtimes as documented in the 2015 NHS England review of the 10 key characteristics of good nutrition and hydration care [10].

There is little high-quality evidence to support that Protected Mealtimes are effective and improve nutritional intake despite the support for the intervention. Seven observational studies [11–17] have described the effect of Protected Mealtimes on the estimated energy intake of patients. These studies have reported various levels of success in implementing Protected Mealtimes, with four studies reporting non-significant increases in energy intake [11, 13–15] whilst energy intake declined in the other three studies [12, 16, 17]. No clinical trials using a high-quality study design have previously evaluated Protected Mealtimes and its impact on nutritional outcomes. This research fills this gap and aimed to measure the effect of implementing Protected Mealtimes within clinical trial conditions on the energy and protein intake of patients admitted to the subacute setting.

## Methods

The full protocol for this stepped wedge cluster randomised trial has been described in detail [18]. The main study features are summarised here.

### Trial design

**Fig. 1:**
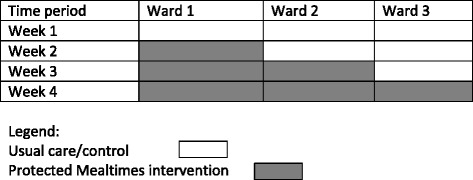
Schematic representation of Protected Mealtimes study design

We undertook a cluster randomised, stepped wedge controlled trial over 4 weeks with three clusters (one cluster = one ward) containing a total of 84 beds. Commencing at week 1, where all clusters were provided with usual care, one cluster crossed from control to intervention every week until all clusters had received the intervention. This trial design is schematically represented in Fig. 1.

This study design was selected for several reasons. There was a desire for all clusters to receive the intervention due to practice guidelines advocating for their use, and the stepped wedge design would allow for ward-level variability in other clinical practices and patient case mix to be accounted for [19]. From a practical perspective, it was also considered unfeasible to roll out the Protected Mealtimes intervention across all hospital wards simultaneously, making the sequential roll-out in a stepped wedge design more viable.

### Study setting and participants

This pragmatic trial aimed to test the effectiveness of the intervention within a service delivery setting. The trial was conducted in a publicly funded healthcare network in Melbourne, Australia, where an extensive range of acute, subacute and ambulatory services were provided. Three different hospital sites with a strong subacute focus were utilised in order to reduce the contamination throughout the implementation period.

All patients were eligible to receive the intervention; only those receiving no oral nutrition or receiving end-of-life care were excluded. The study was conducted via a waiver of consent so that the effect of the intervention on a representative and generalisable patient cohort could be tested. Ethical approval was provided by the relevant Healthcare network (LR69-2014) and University (CF15/414 – 2015000202) human ethics committees.

### Intervention and control conditions

The control condition used ward mealtime processes that were in place as a part of usual care. The healthcare organisation did not have a Protected Mealtimes policy in place previously or at any time during the trial period. An implementation framework was developed based on the Protected Mealtimes Policy of the Hospital Caterers Association [9]; we aimed to implement each aspect of the policy. The effect of the intervention on pre-specified outcomes was evaluated with fidelity of implementation also measured using a pre-determined evaluation plan.

### Procedure

One month prior to the study commencement, the study statistician randomised the order of clusters for implementation and the allocation of patient bed observations using a computer-generated random allocation sequence (https://www.random.org/sequences/). Staff members responsible for assigning patients to beds were blinded to this allocation sequence. Data collectors were provided with the random allocation sequence for the study duration during the trial protocol training session. Neither the order of implementation nor the dates/beds for patient observation were modified from those pre-specified.

Principles of implementation science were applied using the behaviour change wheel [20]. Four main intervention approaches supported this translation project: education, restrictions, environmental restructuring, and enablement. A range of strategies were developed and negotiated with relevant staff to optimise implementation of Protected Mealtimes. These included the following:

1. Education: Training of ward staff (including nursing, medical and allied health staff) and foodservice staff preceding the implementation. Three sessions (each approximately 1 hour in length) were facilitated for all available ward-based clinical staff by the principal investigator on the ward during the week prior to implementation of the intervention.
2. Restrictions: Ward door signage and door closures for mealtimes to promote positive mealtime interactions and minimise unnecessary disruptions. The door signage read: “Food is an important part of hospital treatment for each patient. On this ward, mealtimes occur at 8.00–8:30 am, 12.00–12.30 pm, 5.00–5.30 pm. Please encourage patients to eat their meals if you visit during mealtimes.”
3. Environmental restructuring: A delay of 10 minutes to meal delivery on one ward was facilitated with foodservices staff to enable ward routines to be completed before mealtime. Discussions were held with senior nursing staff regarding the implications of moving medication management from running concurrently with mealtime. The consensus arose that staff would actively endeavour to deliver medications without negatively interrupting meals.
4. Enablement: The pre-existing nutrition executive committee had oversight of the study. The NHS review of Protected Mealtimes [21] identified that lack of “board to ward” level leadership was a key factor influencing implementation. Given that policy development was dependent on the clinical trial findings, we did not implement a policy framework, but instead the trial was supported through this executive committee governance. Data collectors remained blinded to the intervention throughout.

### Outcome measurement

Outcomes and implementation fidelity were measured by 20 data collectors (Nutrition and Dietetic students in year 3 of a 4-year program) who received a full day of specific training prior to the study commencement to augment their prior taught skills in dietary assessment. This training focussed on aspects of outcome and fidelity assessment including estimation of intake using the one-quarter method and mealtime simulations to limit inter-rater variability of interruption data. Validated or standardised tools were used by data collectors to minimise measurement bias. To obtain the primary outcome data of energy and protein intake, observation of actual food and drink consumption occurred at main meals (breakfast, lunch and dinner) and mid-meals (morning and afternoon tea). A pair of data collectors (breakfast/morning tea/lunch and lunch/afternoon tea/dinner) observed and recorded outcome measures of three or four patients per day dependent on the ward geography. The one-quarter portion method [22] was used to estimate consumption of each food item. These estimates were converted to energy (kilojoules/day) and protein (grams/day) using NUTTAB 2010 in Foodworks 7.0 (Xyris software) based on the known nutrient composition of each serve. Data were collected at a per patient per day (e.g. 24-hour food intake, nutritional status, weight); per patient per meal period (e.g. whether patients were positioned upright for eating), and a per interruption (e.g. interruption length and whether interruptions were positive or negative) level.

Participants were weighed by nurses using standing or seated scales. Height was obtained from the medical record; where not recorded, it was measured by data collectors using the measurement of ulnar length and extrapolated to calculate height according to standard methods [23]. Body mass index was calculated. Resting energy expenditure was estimated using an activity factor of 1.42 with no stress factor applied [24]. Requirements for protein intake were estimated at 1.06 g/kg body weight [25]. Nutritional status was determined using the Subjective Global Assessment [26], where a rating of A indicated the absence of malnutrition, whilst B (mildly/moderately) or C (severely) indicated the presence of malnutrition. Hand grip strength was obtained as a secondary measure of nutritional status using defined methods in the non-dominant hand [27], and Functional Independence Measure (FIM)(total and eating subsection) [28] scores on admission and discharge were obtained from the medical record.

### Assessment of fidelity of delivery

Each aspect of the implementation of the Hospital Caterers Association Protected Mealtimes policy was assessed a priori. Methods included the timing of mealtime interruptions using stopwatches, and classifying each interruption as either positive (where eating was encouraged or supported) or negative (i.e. interruptions that hindered food intake).

### Statistical analysis

Descriptive statistics including mean ± standard deviation (SD), median and interquartile range (where appropriate) were calculated in Microsoft Excel. The comparison of primary outcomes between intervention and control periods was undertaken using Stata, release 13 (LP StataCorp, College Station, TX, USA: StataCorp LP, 2014). We examined (1) the change in energy and protein intakes and (2) the change in the deficit (or gain) of energy and protein intakes. A multi-level, mixed effects generalised linear model was used where the week of the study was treated as a categorical fixed factor, and patient and ward were treated as random factors where patients were nested within wards. We undertook a secondary analysis for these comparisons where we accounted for age, nutritional status and type of subacute ward as fixed effect covariates. A significance level of *p* < 0.05 was applied.

### Sample size

**Fig. 2:**
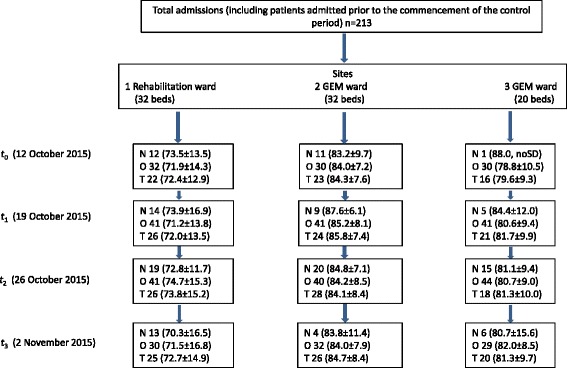
Trial profile. *GEM* geriatric evaluation and management, *N* number of new admissions to unit (mean age in years ± SD), *O* Observations of 24-hour intake (mean age in years ± SD), *T* total number of unique participants (mean age in years ± SD)

This study had the capability to detect a change in 24-hour energy intake of 1900 kJ; this was the energy deficit measured in patients admitted to the same wards in our pilot study [6]. A total number of 84 observations were required to provide 84% power to detect a significant difference of *p* < 0.05 in the primary outcome. An average cluster size of at least seven participants was required; more 24-hour intake measurements were conducted to account for any incomplete records. Repeated measures on some individuals did occur; hence, the participant numbers within each cluster are described within the study flow diagram (Fig. 2). The trial statistical analysis plan was pre-specified [18].

### Deviations from published protocol

The protocol for this trial had included the description of comparing Functional Independence Measure scores and hand grip strength as secondary outcomes. However, we were unable to collect data at transition points between control and intervention periods for our participants. Hence, we have not proceeded with these secondary analyses. Also, the protocol reported that staff training would occur in the 4 days preceding the intervention; due to nursing staff rostering, this occurred during the 3 weekdays prior to the intervention commencement.

## Results

In total, 149 unique individual participants were observed including 38 participants crossing over from the control to intervention period as Protected Mealtimes was implemented. Observations were made of 24-hour food intake on 416 occasions; intake was recorded across 1248 meals and 832 mid-meals. Baseline characteristics are described in Table 1 for the unique individual participants and for the entire sample of participant observations. No participants met the exclusion criteria.

**Table 1:** Baseline characteristics of Protected Mealtimes study participants

Variable	Unique participants		All participant observations	
	Control period *n* = 82	Intervention period *n* = 105	Control period *n* = 210	Intervention period *n* = 206
Age (mean ± SD) (range)	80.5 ± 10.7 (40–99)	78.6 ± 12.9 (23–100)	80.7 ± 10.2 (40–99)	77.9 ± 13.5 (23–100)
Women (*n*; %)	49; 59.8	69; 65.7	148; 70.5	135; 65.5
Men (*n*; %)	33; 40.2	36; 34.3	62; 29.5	71; 34.5
Length of stay (days) (mean ± SD) (range)	54.0 ± 39.7 (9–171)	49.6 ± 37.8 (8–171)	60.7 ± 41.5 (9–171)	52.9 ± 37.3 (9–171)
Primary diagnosis at discharge (*n*): Medical disorders^a^	26	33	65	53
Orthopaedic, musculoskeletal upper limb and lower limb fractures, excluding hip)	14	24	33	51
Hip fracture	9	8	32	18
Stroke	8	13	13	37
Other neurological disease (includes Parkinson’s disease and dementia)	6	10	16	20
Cardiac	5	4	15	6
Respiratory	12	11	32	18
Other surgery	2	1	4	3
Nutritional status: SGA A (well nourished) (*n* (%))	*n* = 81 41 (50.6)	*n* = 101 48 (47.5)	*n* = 208 111 (53.4)	*n* = 200 96 (48.0)
SGA B (mildly or moderately malnourished) (*n* (%))	37 (45.7)	48 (47.5)	92 (44.2)	93 (46.5)
SGA C (severely malnourished) (*n* (%))	3 (3.7)	5 (5.0)	5 (2.4)	11 (5.5)
Body mass index (kg/m^2^): *n* (%)				
<18.5	*n* = 81 10 (12.3)	*n* = 101 17 (16.8)	*n* = 210 22 (10.5)	*n* = 202 28 (13.9)
≥18.5	71 (87.7)	84 (83.2)	188 (89.5)	174 (86.1)
Admission FIM	*n* = 82	*n* = 103	*n* = 207	*n* = 204
Total score (range 18–126) (mean ± SD)	66.0 ± 21.4	66.1 ± 21.5	65.0 ± 19.9	63.1 ± 23.3
Eating component (range 1–7) (mean ± SD)	5.1 ± 1.5	5.2 ± 1.6	5.1 ± 1.4	5.0 ± 1.6
Discharge FIM	*n* = 76	*n* = 100	*n* = 202	*n* = 203
Total score (range 18–126) (mean ± SD)	82.3 ± 25.6	82.7 ± 27.2	83.5 ± 23.3	81.8 ± 28.4
Eating component (range 1–7) (mean ± SD)	5.9 ± 1.4	5.8 ± 1.6	6.0 ± 1.3	5.7 ± 1.6
Hand grip strength (kg) (mean ± SD)	*n* = 75 15.3 ± 9.0	*n* = 95 14.4 ± 8.1	*n* = 187 14.3 ± 7.6	*n* = 186 15.0 ± 9.1
Death during admission (*n*; %)	4; 4.8	2; 1.9	7; 3.4	5; 2.4

*SD* standard deviation, *SGA* Subjective Global Assessment [26], *FIM* Functional Independence Measure [28] of 18 items

^a^Includes cancer, diabetes, complications associated with medical or surgical interventions, infections and general malaise

Table 2 indicates that there was no impact of the intervention on the primary outcomes based on our unadjusted analyses. The energy deficit variable was different between intervention and control periods once age, nutritional status and type of subacute ward were taken into account. This finding indicated that the energy deficit reduced following introduction of the intervention.

**Table 2:** Nutritional outcomes compared between the control and intervention periods

	Control period *n* = 210	Intervention period *n* = 206	Cumulative unit-level effect of intervention over time^a^ (coefficient [robust 95% CI], *p* value)	Intracluster correlation coefficient: unit (95% CI)	Intracluster correlation coefficient: patient within unit (95% CI)	Cumulative unit-level effect of intervention over time^a^ (adjusted coefficient [robust 95% CI], *p* value)	Intracluster correlation coefficient: unit (95% CI)	Intracluster correlation coefficient: patient within unit (95% CI)
Energy intake (kJ/day) (mean ± SD)	6532 ± 2328	6479 ± 2486	-51 (-699 to 597), *p* = 0.876	1.14 × 10^11^ (NA to 1)	0.43 (0.37 to 0.50)	515 (-237 to 1269), *p* = 0.179	2.37 × 10^21^ (2.37 × 10^21^ to 2.37 × 10^21^)	0.31 (0.24 to 0.40)
Protein intake (g/day) (mean ± SD)	67.0 ± 25.2	68.6 ± 26.0	-0.63 (-7.585 to 6.334), *p* = 0.860	1.43 × 10^16^ (1.43 x 10^16^ to 1.43 × 10^16^)	0.005 (3.52 × 10^9^ to 1.00)	2.53 (-5.56 to 10.62), *p* = 0.540	3.06 × 10^14^ (NA to 1)	0.002 (1.47 × 10^18^ to 1)
Energy deficit^b^ (kJ/day) (mean ± SD)	1392 ± 3037	1116 ± 2967	-291 (-1104 to 521), *p* = 0.483	0.04 (0.005 to 0.28)	0.73 (0.65 to 0.79)	-1405 (-2354 to -457), *p* = 0.004	0.015 (0.0003 to 0.44)	0.73 (0.65 to 0.79)
Protein deficit^b^ (g/day) (mean ± SD)	7.1 ± 32.1	2.6 ± 30.1	-0.44 (-6.69 to 5.80), *p* = 0.890	0.01 (0.0002 to 0.38)	0.67 (0.59 to 0.75)	-0.44 (-6.69 to 5.81), *p* = 0.890	1.86 × 10^6^ (NA to 1)	0.33 (0.25 to 0.42)

*SD* standard deviation, *CI* confidence interval;

^a^Data clustered by ward

^b^Deficit calculated as the difference between estimated intake and estimated requirements

Intervention fidelity outcomes (Table 3) indicate that there may have been several areas of practice change attributable to the intervention. There was a 26.2% increase in positive interruptions recorded from the control to intervention period and a 17.6% decrease in negative interruptions. Ward entry doors appeared to be closed and meal signs displayed more frequently during the intervention period. There also appeared to be more nurses and others providing mealtime assistance during the intervention period.

**Table 3:** Fidelity with the Protected Mealtimes intervention

Protected Mealtimes policy component	Control period	Intervention period
Mealtime interruptions:		
Number of positive interruptions	805	1016
Length of positive interruptions (seconds)(median, IQR)^a^	18 (5–50)	20 (8–52)
Reason for positive interruptions; *n* (%)		
Mealtime assistance	167 (20.7)	227 (22.3)
Encouragement with eating	180 (22.4)	268 (26.4)
Re-positioning	124 (15.4)	183 (18.0)
Assistance with opening food packaging	111 (13.8)	176 (17.3)
Other reason	219 (27.2)	158 (15.6)
No reason recorded	4 (0.5)	4 (0.4)
Number of negative interruptions	579	477
Length of negative interruptions (seconds)(median, IQR)	50 (23–132)	54 (23–140)
Reason for negative interruptions: *n* (%)		
Medications	235 (40.6)	206 (43.2)
Ward round	12 (2.1)	1 (0.2)
Patient observations (e.g. temperature, BP)	42 (7.3)	39 (8.2)
Non-essential treatment	68 (11.7)	36 (7.5)
Interview	64 (11.0)	32 (6.7)
Support staff role (e.g. menu completion)	33 (5.7)	26 (5.4)
Telephone call	7 (1.2)	17 (3.6)
Other	115 (19.9)	120 (25.2)
Missing	3 (0.5)	0
Non-nurses providing mealtime assistance (mean per meal)	1.3	2.9
Nurses providing mealtime assistance (mean per meal)	0.9	1.3
Patient required toilet during meals (*n*)	7	15
Observation of patient hand washing prior to food consumption (where hands could be washed) *n* (%)	73 (11.8)	63 (9.9)
Observation of patient in an upright position to eat meal; *n* (%)	591 (95.9)	593 (94.4)
Ability to reach food within 10 minutes of meal commencement; *n* (%)	581 (93.4)	588 (92.5)
Functional Independence Measure completed (of unique participants) *n* (%)	81 (98.8)	104 (99.0)
Meal sign displayed (during meals observed) *n* (%)	13 (48.1)	31 (93.9)
Ward entry doors closed (during meals observed) *n* (%)	9 (33.3)	23 (69.7)
Staff providing mealtime assistance being called to the telephone (*n*)	2	2
Dining room in use	Not measured	No change
Dining room clean and set up for dining	11 of 11	12 of 12

*PM* Protected Mealtimes, *IQR* Inter-quartile range, *BP* blood pressure

^a^Length of interruptions was calculated among only those participants experiencing positive or negative interruptions

## Discussion

The unadjusted analyses did not identify any statistically significant changes in energy or protein intake as a result of implementing the Protected Mealtimes intervention. These results are similar to findings of several other observational studies of Protected Mealtimes implementation, where no significant difference has been recorded in either energy [11–13, 16, 17] or protein intake [13, 16, 17]. These previous studies had not reported the estimated energy or protein requirements of participants or other measures of nutritional status, marking an important methodological advance in the present study. Our secondary analyses accounting for age, nutritional status and type of subacute ward identified a significant reduction in the gap between estimated energy intake and energy requirements; however, with energy deficit not reported in any previous studies of Protected Mealtimes, it is difficult to place this result into a broader context.

Informed by the seven previous observational studies and the results of this clinical trial, the evidence that implementation of Protected Mealtimes will improve nutritional outcomes for patients remains unproven. Within the multitude of system and staff changes needed to implement Protected Mealtimes across a hospital setting, determining which features of the approach have effect and which do not may assist in defining the way forward for practice. Certain features have previously been individually identified as having a statistically significant effect on food intake. These include providing help when there is a documented need for mealtime assistance, introducing mealtime volunteers whose role it is to assist patients to eat and drink, providing time for patients to eat and appropriate mealtime positioning to enable patients to eat and drink safely [29]. These seem like simple steps that are helpful and proven effective.

A recent systematic review with meta-analyses of the effect of implementing mealtime assistance programs [30] showed statistically significant improvements for energy and protein intake of patients. This approach is likely considerably easier to implement than a whole Protected Mealtimes policy, as it directs assistance to those who specifically need it. Other food-based approaches [31] and implementation of oral nutrition support products in addition to oral nutritional intake [32] have also been shown to increase the energy and protein intake of hospitalised patients. All of these approaches require defined pathways to identify patients who are malnourished or at risk of malnutrition (e.g. nutrition screening, rescreening and assessment) and those who require feeding assistance with subsequent management protocols. They may require less organisational change than the broad systems approach of Protected Mealtimes, and they have been shown to deliver enhanced patient outcomes. Other positive outcomes may arise from the implementation of Protected Mealtimes, including an increased inter-professional focus on nutrition throughout the ward, and improved quality of life for patients through decreased mealtime interruptions. These aspects were not evaluated within this trial, nor have they been evaluated by other researchers.

### Limitations

Although conducted over a relatively short period and with practice changes informed by the implementation science literature, there was variability in implementation across different aspects of Protected Mealtimes policy components. As suggested by fidelity measurements, Protected Mealtimes differed in the extent of implementation. Further, the relatively short implementation timeframe did not enable longitudinal measures of nutritional status to be recorded. As with all studies of nutritional intake, some error in the estimate of dietary intake and conversion of these data to nutrients is acknowledged [33]. Error may also have been introduced through the estimate of nutritional requirements of individuals and inter-observer variation. Further, there may have been variability in the measurement of fidelity with the intervention (e.g. judgements of whether mealtime interruptions were positive or negative); this was reduced through the pre-study training program for observers and testing of their ability to record observations accurately.

## Conclusions

This trial has used a high-quality study design to implement Protected Mealtimes in the clinical setting. No significant changes resulted in the energy or protein intakes of patients, consistent with the findings of previous observational studies. The reduction in the energy deficit associated with intervention identified in the secondary analysis suggests that further exploration of the approach is warranted, but perhaps more direct attention to feeding patients who need assistance would provide impact without large-scale organisational systems change. In the absence of a strong evidence across the primary analysis, we encourage clinicians to consider other evidence-based approaches for the prevention and treatment of malnutrition in hospitalised patients, as there is no doubt that there is a need for action. Further research will determine the extent of other important benefits that may arise from Protected Mealtimes implementation, such as patient experience and satisfaction.
